# Oral Ketamine-Assisted Psychotherapy Leading to Prolonged Remission in Recurrent Dissociative Neurological Symptom Disorder With Mutism: A Case Report

**DOI:** 10.7759/cureus.117827

**Published:** 2026-10-12

**Authors:** Anirudh Bakam

**Affiliations:** 1 Psychiatry, All India Institute of Medical Sciences, Bibinagar, Hyderabad, IND

**Keywords:** abreaction, dissociative disorder, functional mutism, functional neurological disorder, ketamine-assisted psychotherapy, lorazepam, psychodynamic psychotherapy

## Abstract

Dissociative neurological symptom disorder (DNSD), termed functional neurological symptom disorder in the Diagnostic and Statistical Manual of Mental Disorders, Fifth Edition, Text Revision (DSM-5-TR), presents with neurological symptoms not explained by structural disease. The first line treatment of DNSD is psychotherapy, but when the conflict maintaining the symptoms cannot be put into words, psychotherapy often stalls. This report describes a 56-year-old woman with a four-year history of episodic loss of consciousness and progressive speech loss, with normal neurological investigations. Escitalopram, intravenous ketamine infusions given without psychotherapy, and venlafaxine produced no meaningful benefit. A 30-day inpatient program of psychotherapy, family therapy, and speech therapy restored her speech, but she relapsed into complete mutism four months after discharge, and no stressor could be identified. A lorazepam-assisted interview produced sedation without speech. She then received six alternate-day sessions of oral ketamine-assisted psychotherapy, titrated from 50 mg to 150 mg, within a psychodynamically informed frame; while venlafaxine 225 mg/day was continued and family therapy was delivered alongside. She spoke a few words in the third session (100 mg) and conversed spontaneously in the fourth (150 mg), describing a conflict between a rigid moral self-image of never being greedy and unspoken distress over a family property dispute. Speech has been sustained for more than one year, and all psychotropic medication has since been withdrawn. The report proposes that, this single case is hypothesis-generating. It suggests that ketamine may be a useful and a better abreactive agent in selected patients with treatment-resistant DNSD, and that the therapeutic frame is also essential for the improvement to happen, and further controlled comparison with benzodiazepine-assisted interviews is warranted in the future.

## Introduction

Dissociative neurological symptom disorder (DNSD), classified in the International Classification of Diseases, 11th Revision (ICD-11) under dissociative disorders, is characterized by motor, sensory, speech, or cognitive symptoms that are not consistent with a recognized disease of the nervous system [1]. The Diagnostic and Statistical Manual of Mental Disorders, Fifth Edition, Text Revision (DSM-5-TR) describes the same condition as functional neurological symptom disorder (conversion disorder) and requires clinical findings that show incompatibility between the symptom and recognized neurological or medical conditions [2]. In this report, dissociative (functional) mutism refers to loss of the ability to speak, with preserved comprehension, that is not explained by structural disease. Functional symptoms are a common reason for neurological consultation. In a prospective study of 3,781 consecutive new patients attending neurology clinics in Scotland, functional and psychological symptoms accounted for 16% of referrals [3]. Contemporary accounts describe changes in attention, expectation, emotional processing, and sense of agency [4], and treatment usually combines psychoeducation, psychological therapy, and physical or speech rehabilitation, depending on the symptoms [5].

The psychodynamic account remains clinically useful. It holds that the symptom gives bodily form to a conflict that the person cannot consciously acknowledge. Classical psychoanalysis, the method developed by Freud, relies on free association and interpretation within a regular, structured setting so that unconscious conflict can gradually enter awareness. Abreaction refers to the discharge of emotion that occurs when a warded-off memory or conflict is recalled and expressed, and in this tradition it was regarded as a step toward resolution. Dissociative mutism makes this approach far more difficult. If the conflict cannot be put into words, a therapy that depends on words has little to work with, and a patient who cannot speak cannot even begin.

Drug-assisted interviews were developed partly for this problem alongside narcoanalysis for forensic reasons. In 1930, Bleckwenn reported that intravenous sodium amobarbital produced a lucid interval in which mute catatonic patients could converse, and the amobarbital interview was soon applied to conversion and dissociative states [6]. In practice, a sedative is injected slowly while the clinician talks with the patient; at the point of mild drowsiness, before sleep, the patient is encouraged to recall distressing events, express emotion, and attempt the lost function, usually with positive suggestion of recovery [6]. Benzodiazepines later largely replaced barbiturates because of their greater safety [6]. A systematic review of abreaction in conversion disorder found no controlled studies; across the available case reports and series, emotional catharsis and suggestion during the interview were associated with recovery, and there was insufficient evidence to favor any particular drug [7]. The method has recently been proposed as worth revisiting [8].

Ketamine entered psychiatry partly as an abreactive agent. In 1973, Khorramzadeh and Lotfy used it to facilitate psychotherapy in psychiatric patients, including those with conversion and dissociative reactions, most of whom improved [9]. In 1986, Golechha and colleagues gave intramuscular ketamine (0.5 to 1.5 mg/kg) to 30 patients requiring narcoanalysis and compared it with thiopental and amobarbital-methamphetamine interviews. They concluded that ketamine was an effective abreactant with better cardiorespiratory safety and easier administration, although symptom outcomes were not reported in detail [10]. Then there is a large gap in literature in between on ketamine. More recently, a 15-year-old boy with dissociative amnesia, including loss of three languages, recovered after a single session of intramuscular ketamine abreaction at 1 mg/kg [11].

A 2026 systematic review identified 13 studies of ketamine or esketamine in functional neurological disorder. Nearly all were single case reports or small uncontrolled series that differed in agent, route, dose, and outcome measures, and the authors concluded that further study is justified but that efficacy cannot yet be established [12]. One included report described oral ketamine-assisted therapy at 100 to 150 mg per session for refractory functional seizures [13]. Ketamine-assisted psychotherapy (KAP) combines subanesthetic doses of ketamine, an N-methyl-D-aspartate (NMDA) receptor antagonist, with psychotherapy before, during, and after dosing. It has been studied mainly in depression, post-traumatic stress disorder, and substance use, where it is thought to improve synaptic plasticity and thereby enhancing learning during the process of therapy [14].

The current report describes a woman with recurrent DNSD and complete mutism in whom a lorazepam-assisted interview failed and oral KAP, delivered within a psychodynamically informed frame, was followed by return of speech and sustained remission without any psychotropic medication. Because both drug-assisted approaches were tried in the same patient during the same relapse, the case offers an unusual within-patient comparison, although an uncontrolled one. A search of PubMed and Google Scholar up to January 2026, using combinations of the terms "ketamine," "abreaction," "narcoanalysis," "drug-assisted interview," "functional neurological disorder," "conversion disorder," "dissociative neurological symptom disorder," "mutism," and "aphonia," identified no previous report of oral KAP for dissociative mutism or of ketamine succeeding after a failed benzodiazepine interview in the same patient.

## Case presentation

A 56-year-old married woman, a graduate from an urban area of India, was referred to a tertiary neuropsychiatric institute with a four-year history of illness. The illness began acutely with continuous dizziness through the day and episodes of loss of consciousness lasting 20 to 30 minutes. Each episode was followed by 30 to 40 minutes in which she did not recognize her surroundings, and then by full recovery. There were no falls, injuries, abnormal movements, incontinence, or focal weakness. During the episode patient would be unresponsive throughout, and even after complete recovery she would not be aware of what had just happened with her. Brief periods of speech loss, in which she could produce only sounds such as "aa" or "uu," also accompanied the episodes. The episodes were infrequent over the first year, then increased to one or two per day over the three years before presentation. Her speech loss progressed, and about six months before presentation, she became completely mute. No triggers were apparent.

During the first two years, she consulted several physicians and neurologists. Repeated blood investigations, magnetic resonance imaging of the brain, electroencephalography (EEG), and video electroencephalography were normal. During one of the EEG recordings, tuning fork induction produced a similar episode of loss of consciousness, followed by confusion and speech difficulty, without any corresponding EEG changes. She received levetiracetam 1,000 mg/day for about a year without benefit, and it was tapered when she was referred for private psychiatric care three years after onset (about one year before presentation). Her family also sought help at temples during this period. During one faith-healing session, she lost consciousness and, with her eyes closed, spoke as if possessed by a goddess, saying that she would not recover until the end of the following year and would suffer until then. Religious rituals were then performed at home, without change.

Three years after onset, a psychiatrist diagnosed DNSD and started escitalopram, which was increased to 20 mg/day and continued for three to four months without benefit. Her symptoms worsened after the death of a distant cousin. She then received six outpatient intravenous ketamine infusions of 0.5 mg/kg, each given over 30 mins, without any co-administered sedatives. These details were obtained from the doctors medical records. The records stated that the infusions were given for depressive symptoms, although no depressive syndrome was evident when we later assessed her. She slept through each infusion, no psychotherapy was offered during them, and there was no benefit. About six months before presentation, when she had become completely mute, the private psychiatrist admitted her and replaced escitalopram with venlafaxine. Individual therapy was attempted but was limited by her mutism. When electroconvulsive therapy was suggested, the family declined and sought a second opinion, which led to referral to the tertiary institute.

She was admitted there for 30 days. This was the first contact of her with us. She was well kempt, cooperative, and anxious. She was completely mute but followed spoken instructions and communicated fluently by writing in Telugu. Despite not able to speak, she was able to make whispering jargon sounds (“aah”, “uuu” etc.,) and was able to cough normally. Neurological examination was otherwise normal. She repeatedly wrote that she was preoccupied with her loss of speech and fainting episodes.

The diagnosis of DNSD was re-established based on ICD-11 [1,2]. Several differential diagnoses were considered. Organic aphonia was considered unlikely because cough was preserved and laryngoscopy was normal. A neurodegenerative aphasia, such as primary progressive aphasia, was unlikely because her written language was intact, brain imaging was normal, and her speech later recovered fully. Epilepsy was considered unlikely because of the long duration of the episodes, the absence of motor features, injury, or incontinence, the normal electroencephalographic studies (and the capture of a typical episode without epileptiform change), and the lack of response to levetiracetam. Cardiac syncope was considered unlikely because the episodes lasted far longer than typical syncope and caused no falls or injury, and cardiac evaluation was normal. Possession trance disorder [1] was considered because of the possession-like episode during the faith-healing session, but this occurred only once, in a ritual setting in which such experiences are culturally sanctioned, and did not recur.

Venlafaxine was increased to 225 mg/day for anticipatory anxiety about the episodes. Individual psychotherapy combined supportive and cognitive-behavioral elements: rapport building and validation through written communication, psychoeducation using an explanatory model of functional symptoms, guided written exploration of her life history and relationships, and cognitive work on catastrophic beliefs about the episodes was done. The first days were spent building rapport and validating her distress, which she wrote about repeatedly. She was then encouraged to write about other parts of her life. On day four, she wrote at length about a comfortable childhood, her husband's limited involvement in family matters, and a quarrel with her sister during her daughter's wedding, after which the sister stopped speaking to her. Around day 10, she briefly wrote about an unresolved partition of family property between her husband and his sisters. However, she repeatedly wrote that these events did not affect her much.

Individual therapy was combined with family therapy, in which her husband and son received psychoeducation about the illness and learned differential reinforcement (attending to and encouraging healthy behavior while giving less attention to symptoms). She also received speech therapy (with graded tasks such as humming, sighing, and vowel production). She accepted the psychological model of her illness only reluctantly. After about day 20, as rapport improved, her dizziness and episodes decreased, and she began speaking during speech therapy for the first time in about six months. Family work then focused on increasing her husband's and son's involvement in family decisions. She was discharged on day 30 in remission on venlafaxine 225 mg/day, with local follow-up for individual and family therapy.

About four months after discharge (five months after presentation), both symptoms returned without an apparent stressor. She again became completely mute and had one or two episodes of loss of consciousness daily, and she remained mute for the next three months. When asked in writing, she wrote that there was no stressor she could consciously think of and that she did not know why the symptoms had returned. Two or three further sessions produced no progress. Seven months after presentation, a lorazepam-assisted interview was attempted: 1 mg of lorazepam diluted in 100 mL of normal saline was infused intravenously over 10-15 minutes, while the therapist encouraged her to speak. Even at this dose, she became markedly drowsy and fell asleep. On waking, she was as before and did not speak despite repeated suggestion.

Because conventional approaches had failed to reach what was maintaining the symptoms, outpatient KAP was planned at my center later that month, and written informed consent was obtained. Sessions were held on alternate days. In each session, she sat in a reclining chair and sipped oral ketamine mixed in bottled mango juice over one hour. The dose was 50 mg in session one, 100 mg in sessions two and three, and 150 mg in sessions four to six. Her body weight was 71 kg, so the doses corresponded to approximately 0.7 to 2.11 mg/kg. Monitoring: vital signs before, during, and after each session; post-session observation period; discharge home with an escort. All doses were taken under direct supervision in the clinic, and none were dispensed for home use. She was semi-drowsy during the sessions. The therapist stayed with her throughout, asked her not to fall asleep, talked with her about her day, encouraged her to attempt speech despite the difficulty, and explored possible internal conflicts with open questions. Illness severity was rated by the treating clinician before KAP and at each session using the Clinical Global Impression-Severity (CGI-S) scale, a seven-point measure ranging from 1 (normal, not at all ill) to 7 (among the most extremely ill patients) [15]. Her CGI-S score was seven at baseline and at the first session, and decreased to one by the sixth session. The sessions are summarized in Table 1.

**Table 1:** Oral ketamine-assisted psychotherapy sessions CGI-S: Clinical Global Impression-Severity (1, normal; 7, among the most extremely ill). Baseline CGI-S before KAP was 7.

Session	Oral dose	Main therapeutic process	CGI-S score
Day 1	50 mg	Neutral conversation about positive experiences; no speech	7
Day 3	100 mg	No speech; distress about inability to speak expressed through gestures; empathic validation	7
Day 5	100 mg	First few words after three months of mutism; continued validation and exploration	6
Day 7	150 mg	Spontaneous speech at 10 to 15 minutes; disclosure of the property conflict and moral self-image; emotional ventilation	3
Day 9	150 mg	Sustained speech; supportive psychotherapy and cognitive restructuring	2
Day 11	150 mg	Sustained speech; family session with husband on open communication and differential reinforcement	1

In session three, at 100 mg, she spoke her first few words in three months. In session four, at 150 mg, 10 to 15 minutes after she began sipping, she began speaking spontaneously and answering questions. Asked about stressors, she said she was a moral, hardworking person who had never been greedy. Asked how this related to her present situation, she explained that her mother-in-law wanted to give her husband's house to his sister and had recently written a will to that effect. Her husband, she said, was naive and would not raise the matter, and if she raised it herself, people would call her greedy. This left her in conflict over the matter. The therapist validated her feelings, offered perspective, and allowed her to express the anger and distress she had held back. Session five continued the supportive work, with cognitive restructuring of her belief that asserting a legitimate claim meant being greedy. Session six was a family session, in which her husband was encouraged to communicate openly and to take a firmer, more present role in the family, and differential reinforcement was revisited.

Speech continued through sessions five and six and was sustained thereafter, and the episodes of loss of consciousness stopped. No major adverse effects were observed during or after the sessions, apart from mild dizziness, drowsiness, and nausea. Her husband became more actively involved in family matters. At monthly follow-up, venlafaxine was tapered gradually and stopped, along with all other psychotropic medication. She has remained in remission, speaking normally and without episodes, for about 14 months after KAP.

## Discussion

This patient had DNSD with complete mutism that relapsed after an initially successful multidisciplinary admission. During the relapse, she could not identify a stressor, could not speak, and did not respond to a lorazepam-assisted interview. Oral KAP was followed within four sessions by spontaneous speech, disclosure of a specific conflict, and remission that has lasted more than one year without medication. Three features distinguish this report from earlier ones [9-13]: repeated, titrated oral dosing within an explicitly psychodynamic frame; functional mutism as the target symptom; and an uncontrolled within-patient contrast with a benzodiazepine interview that failed during the same relapse.

One plausible reading of her disclosure follows a classical formulation. Her sense of herself as moral and free of greed was central to her identity. Her mother-in-law's will threatened her family's home, and the legitimate response, to object, would in her social world mark her as the kind of person she had always refused to be. On this reading, the conflict was not consciously available to her, and the symptom carried it in condensed form: she could not speak, and so could not voice the claim she felt she was not allowed to make, and the same silence protected her from the judgment she feared. An alternative reading is equally possible. Her written statement that she could think of no stressor may reflect conscious reluctance to disclose a socially sensitive family matter rather than a lack of access to it. The two readings are not mutually exclusive, and ketamine may have helped in either case by lowering defensiveness. During the first admission, she had briefly written about the property partition while denying its importance, but the specific wish and its forbidden character emerged only under ketamine. The psychodynamic model and contemporary network models [4] can be seen as describing the same clinical event at different levels; in predictive processing terms, the symptom may reflect a strong, rigid prior about the self and the body that ordinary experience fails to update [16].

A cultural formulation adds to this understanding [2]. In many Indian joint families, property passes through the male line and is often settled informally by the senior generation, and a daughter-in-law who openly contests a parent-in-law's decision risks being seen as greedy and disloyal and as bringing shame on the family. Her distress therefore had no socially acceptable verbal outlet. In this setting, bodily and dissociative symptoms, including fainting, loss of speech, and possession states, are recognized idioms of distress that communicate suffering without open confrontation. The possession-like episode during faith healing, and the family's turn to temples before psychiatry, fit this pattern. The family session in which her husband took a firmer role may have worked partly because it resolved the problem within accepted family roles.

Benzodiazepines and barbiturates are the traditional agents for abreaction, and benzodiazepines are generally preferred for their safety [6,7]. In this patient, 1 mg of lorazepam moved her from wakefulness to sleep with no intermediate state in which she could talk. One explanation lies in how gamma-aminobutyric acid (GABA)-A potentiation works: anxiolysis, sedation, and hypnosis lie on a single continuum, and in a sensitive patient the window between reduced inhibition and sleep can be narrow. Ketamine acts differently. As an N-Methyl-D-aspartic acid (NMDA) receptor antagonist at subanesthetic doses, it produces dissociative changes in perception and sense of self while largely preserving arousal and responsiveness [17], and patients receiving KAP describe reduced defensiveness and easier access to emotional material [14]. A patient can therefore be altered but awake, which is the state an abreactive interview aims for. However, this comparison must be interpreted cautiously. The lorazepam interview was a single fixed-dose attempt, conducted at a different time and under different conditions, and the dose was not titrated.

Her earlier intravenous ketamine infusions, given elsewhere are informative. She had already received ketamine without benefit and slept through those sessions. The oral doses used here fall within the range of 50 to 300 mg per occasion reported for oral ketamine in psychiatric practice, and only about 20% to 25% of an oral dose is thought to reach the systemic circulation [18]. The effective sessions are therefore unlikely to have involved greater drug exposure than the failed infusions. What differed was delivery and frame. Slow sipping over an hour produced a gradual rise in drug levels, the therapist kept her awake and engaged, and each session had a clear psychotherapeutic purpose; the intravenous sessions had none of these. This suggests that altered consciousness with preserved responsiveness is useful only when combined with active therapeutic work, consistent with the emphasis on preparation, in-session support, and integration in KAP practice [14,19]. It also argues against attributing her improvement simply to an antidepressant effect of ketamine.

Disclosure alone rarely produces lasting change, and the drug interview was originally intended as the first step of treatment rather than the whole of it [6,7]. Here, sessions five and six were used for working through, and the family session changed the situation that had produced the conflict: her husband took a more active role, so the claim she could not make was, in effect, made on her behalf. The earlier admission had also built rapport and introduced the psychological model, and this groundwork probably contributed. As noted above, the 2026 systematic review found that most reports of ketamine in functional disorders involved esketamine for comorbid depression or single parenteral doses, and only a few used ketamine specifically as an abreactive agent [12]. The present case adds oral dosing, repeated sessions with gradual titration, and a failed benzodiazepine interview in the same patient.

The oral route has practical advantages in outpatient settings where intravenous access and anesthesia support are limited, but its safety needs to be considered carefully. A systematic review of oral ketamine found it generally well tolerated in the available studies, with mostly mild and transient adverse effects, but the evidence base was small, and the authors cautioned about the risks of misuse and diversion [20]. Bioavailability varies between individuals and has been poorly studied [18]. In clinical practice, KAP has been delivered in outpatient offices under clinician supervision [19]. In this case, every dose was taken in the clinic under direct observation, no doses were dispensed for home use, the course was limited to six sessions with close monitoring. Mild dizziness, drowsiness, and nausea were the only adverse effects. These safeguards seem essential if the approach is used elsewhere.

These observations may help clinicians considering drug-assisted interviews in treatment-resistant DNSD. When a benzodiazepine produces sleep rather than speech, this should not be taken as evidence that abreaction cannot work. A randomized comparison of ketamine-assisted and benzodiazepine-assisted interviews within the same psychotherapeutic protocol, with titrated dosing of both agents, would test whether the difference seen here reflects the drug. Outcomes could include return of function, episode frequency, Functional neurological disorder (FND)-specific measures, and systematic ratings of dissociation during sessions.

This report has limitations. It describes a single case, and the improvement cannot be attributed to ketamine with confidence. The lorazepam interview was a single, untitrated attempt under different conditions. Venlafaxine was continued during KAP and withdrawn only afterward, and family therapy was delivered alongside it. Outcomes were limited to the return of speech and a single clinician's Clinical Global Impression-Severity (CGI-S) ratings; episode frequency was not systematically recorded, and dissociation could not be rated with a standardized scale. Expectancy, therapist suggestion, the natural fluctuation of functional symptoms, and possibly the faith-healer's prophecy that she would not recover until the end of the following year may all have contributed.

## Conclusions

In this patient with recurrent DNSD and complete mutism, a lorazepam-assisted interview produced only sleep, and oral ketamine-assisted psychotherapy was followed by spontaneous speech, disclosure of a previously unspoken conflict, and sustained remission off medication. The most defensible lesson is that the therapeutic frame is essential: unframed intravenous ketamine had failed, whereas the same drug given within active psychotherapy was followed by recovery. Whether ketamine is better suited than benzodiazepines to drug-assisted interviews remains a hypothesis that this single case cannot confirm. Controlled comparisons in treatment-resistant DNSD are warranted.
